# High Bandwidth Synaptic Communication and Frequency Tracking in Human Neocortex

**DOI:** 10.1371/journal.pbio.1002007

**Published:** 2014-11-25

**Authors:** Guilherme Testa-Silva, Matthijs B. Verhoog, Daniele Linaro, Christiaan P. J. de Kock, Johannes C. Baayen, Rhiannon M. Meredith, Chris I. De Zeeuw, Michele Giugliano, Huibert D. Mansvelder

**Affiliations:** 1 Department of Integrative Neurophysiology, CNCR, VU University Amsterdam, The Netherlands; 2 Department of Neuroscience, Erasmus Medical Center, Rotterdam, The Netherlands; 3 Netherlands Institute for Neuroscience, Royal Netherlands Academy of Arts and Sciences (KNAW), Amsterdam, The Netherlands; 4 Department of Biomedical Sciences, University of Antwerp, Belgium; 5 Department of Neurosurgery, VU University Medical Center, Neuroscience Campus, Amsterdam, The Netherlands; 6 Department of Computer Science, University of Sheffield, United Kingdom; 7 Brain Mind Institute, Swiss Federal Institute of Technology of Lausanne, Switzerland; Hebrew University, Israel

## Abstract

Neuronal firing, synaptic transmission, and its plasticity form the building blocks for processing and storage of information in the brain. It is unknown whether adult human synapses are more efficient in transferring information between neurons than rodent synapses. To test this, we recorded from connected pairs of pyramidal neurons in acute brain slices of adult human and mouse temporal cortex and probed the dynamical properties of use-dependent plasticity. We found that human synaptic connections were purely depressing and that they recovered three to four times more swiftly from depression than synapses in rodent neocortex. Thereby, during realistic spike trains, the temporal resolution of synaptic information exchange in human synapses substantially surpasses that in mice. Using information theory, we calculate that information transfer between human pyramidal neurons exceeds that of mouse pyramidal neurons by four to nine times, well into the beta and gamma frequency range. In addition, we found that human principal cells tracked fine temporal features, conveyed in received synaptic inputs, at a wider bandwidth than for rodents. Action potential firing probability was reliably phase-locked to input transients up to 1,000 cycles/s because of a steep onset of action potentials in human pyramidal neurons during spike trains, unlike in rodent neurons. Our data show that, in contrast to the widely held views of limited information transfer in rodent depressing synapses, fast recovering synapses of human neurons can actually transfer substantial amounts of information during spike trains. In addition, human pyramidal neurons are equipped to encode high synaptic information content. Thus, adult human cortical microcircuits relay information at a wider bandwidth than rodent microcircuits.

## Abstract 2

Because of fast recovery from synaptic depression and fast-initiated action potentials, neuronal information transfer can have a substantially higher bandwidth in human neocortical circuits than in those of rodents.

## Author Summary

Our ability to think, memorize information, and act appropriately depends on circuits of connected neurons in the brain. In these circuits, neurons pass information to each other using electric pulses (action potentials) that cause the release of chemical neurotransmitters, which alter the membrane electric potential of receiving neurons. Based on the inputs neurons receive, they decide whether to transmit action potentials to other neurons in the circuit to pass on information. During sequences of repeated information transfer, synaptic connections between two neurons temporarily become weaker by synaptic depression. Our knowledge of neuronal information transfer is based on rodent neurons. The properties of synaptic information transfer and synaptic depression in humans are not known. Here, we show that adult human neurons can transfer information with up to ten times higher rates than mouse neurons, because of a three to four times faster recovery from depression. Furthermore, we found that human neurons can respond faster to synaptic inputs, owing to faster initiation of action potentials. Human neurons can thereby reliably encode high input frequencies in their output. Thus, neuronal information transfer can have a substantially higher bandwidth in human neocortical circuits than in rodent brains.

## Introduction

Human cognitive abilities clearly stand out from those of other mammals [1]. Evolutionary development of brain size, encephalization, neocortical thickening, and specialization of cortical circuits [2],[3] most likely underlie the superior human mental capacity, but other factors may contribute as well [4]. Cognitive functions rely on appropriate relay and filtering of information and on efficient communication between brain areas. Ultimately, neuronal firing and synaptic transmission between neurons form the building blocks for coding, processing, and storage of information in the brain [5]. Synapses in particular are fundamental computational units [6]–[8], and the increased complexity of synaptic protein networks was recently put forward as a potential correlate of mammalian cognitive ability [9]–[11]. Given the vast number of synapses in the brain, in the order of a trillion per cubic centimeter [12], even a slight increase in efficacy of synaptic information processing could potentially translate into a substantial elevation of the brain's overall computational performance [13]. Whether human synapses are more efficient in transferring information between neurons is not known and has not been tested directly.

Here, we addressed this question and studied the properties of signal transfer at unitary synaptic connections between pyramidal neurons of adult human and mouse neocortex. We then applied an information theory approach to calculate synaptic transfer performance [7],[14],[15]. We focused on the short-term dynamics of transmission, as synapses are not passive conveyers of information. Instead, they display prominent use-dependent plasticity, which has important roles in information processing [8],[16]. Following chemical signal transduction at a single synapse, postsynaptic signals appear as selectively filtered versions of the train of action potentials (APs) that the presynaptic neuron generates [17],[18]. Amplitudes of successive postsynaptic potentials are in fact transiently and reversibly attenuated or amplified by the context of previous pre- and postsynaptic activation. Whether or not a postsynaptic neuron fires in response to an individual presynaptic AP thus depends on the instantaneous AP frequency, on the short-term dynamical properties of each synapse, and on the previous history [8],[17],[19],[20]. We found that human cortical synapses recover faster from depression than rodent cortical synapses, resulting in a substantially higher information transfer rate than in rodent synapses. In addition, we directly observed that human pyramidal neurons are equipped to encode such a high information content synaptic transmission in their output, unlike rodent pyramidal neurons, by their dynamical excitability properties.

## Results and Discussion

**Figure 1:**
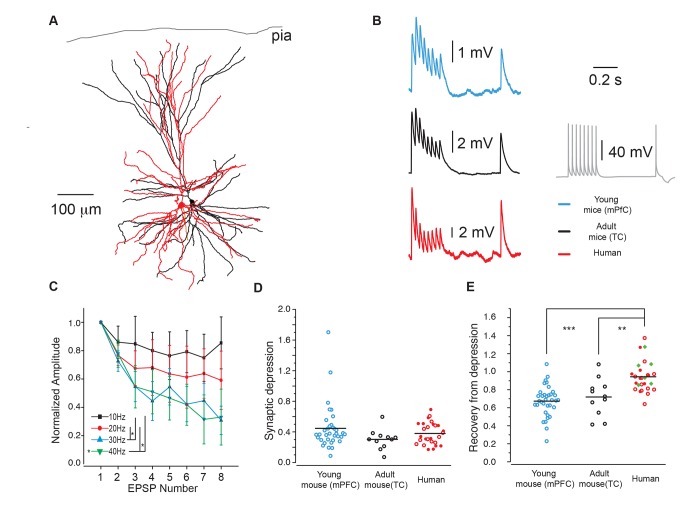
Synapses in the adult human neocortex rapidly recover from depression. (A) Digital reconstruction of a biocytin-filled, synaptically connected pair of layer 2/3 pyramidal neurons in human temporal cortex. (B) Experimentally recorded EPSPs, generated by presynaptic timed APs at 30 Hz followed by a recovery pulse, 500 ms after the 8th pulse. Traces in blue are from young murine medial prefrontal cortex (mPFC) (P12–36), black, from murine temporal association cortex (10–11 weeks) and red from human temporal cortex. Grey is an example of a train of human presynaptic APs. Examples are averages of 30 repetitions. (C) Activity dependence of human short-term synaptic depression. Normalized average EPSPs (three pairs) generated in response to different frequencies (10–40 Hz). (D) Ratio 8th/1st EPSP (mean ± SEM) 0.38±0.03 human, 0.44±0.05 for young mouse synapses and 0.30±0.04 for adult mouse synapses (*p*>0.05). (E) Ratio 9th/1st EPSP (mean ± SEM) 0.94±0.03 for human, whereas young mouse EPSPs were still depressed 0.67±0.03 (*p*<0.001) and adult mouse EPSPs 0.72±0.06 (*p*<0.001). Difference between young and adult mouse ratios were not significant (*p* = 0.7). Human *n* = 27 (14 from tumor patients and 13 from epilepsy patients, see Materials and Methods); young mouse *n* = 35; adult mouse *n* = 11. Filled red circles, human tumor patients; open red circles, human temporal lobe epilepsy patients (300 ms interval between 8th and 9th EPSP). Open blue circles, young mouse group p12–p36; open black circles, adult mouse group 8–11 weeks. Filled green diamonds, human patients with 500 ms interval between 9th and 8th EPSP. (Data deposited in the Dryad repository, http://doi.org/10.5061/dryad.3723p [56]: Data of neuronal reconstructions in Figure 1A.rar; Raw data of human and rodent EPSPs in 30 Hz_EPSPs.rar; Numerical data that generated (B–E) as well as Figure S1A–S1D in Data S1).

In the rodent brain, unitary connections between neocortical pyramidal neurons show frequency-dependent short-term synaptic depression, in response to a sequence of APs [17],[21],[22]. To test whether excitatory connections between adult human neocortical pyramidal neurons show short-term plasticity, and whether this quantitatively resembles that in mouse neocortex, we made whole-cell recordings from synaptically connected layer 2/3 pyramidal neurons of non-pathological samples of cortex from adult human patients (see Materials and Methods) (Figure 1A; Tables S1, S2, S3) [23],[24] and mouse neocortex (medial prefrontal cortex of young mice of 12–36 days old, and temporal association cortex of adult mice of 8–11 weeks old). Human monosynaptic connections showed no facilitation but only frequency-dependent depression, whose occurrence resembled that of mouse synapses (Figure 1A–1D). The amount of depression during a 30 Hz presynaptic AP train did not differ between human and mouse synapses (Figure 1D) (ratio last/first excitatory postsynaptic potential [EPSP]; mean ± standard error of the mean [SEM]: 0.38±0.03 human, 0.44±0.05 for young mouse synapses, and 0.30±0.04 for adult mouse synapses) (*p*>0.05). However, at 0.5 seconds following the end of the AP train, the amplitude of human EPSPs had recovered to the level of the first EPSP in the train (Figure 1B and 1E; ratio recovery/first EPSP 1.01±0.07 [500 ms between recovery and first, *n* = 6, green filled diamonds]); whereas mouse EPSPs were still significantly depressed (young 0.67±0.03; adult 0.72±0.06, *p*<0.001). Moving the ninth AP closer in time to the AP train in the recordings from human unitary connections showed that already after 0.3 seconds the amplitude of the human EPSP had nearly recovered to the level of the first EPSP (Figure 1E; ratio recovery/first EPSP 0.92±0.04; circles).

**Figure 2:**
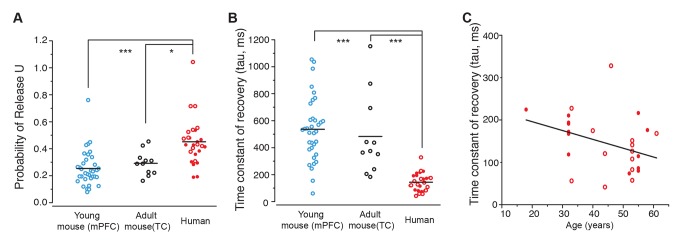
Tsodyks-Markram model for dynamic synapses. (A) Utilization of absolute synaptic efficacy (mean ± SEM), proportional to the probability of release. 0.45±0.03 in human (*n* = 27, 14 from tumor patients and 13 from epilepsy patients) versus 0.25±0.02 and 0.29±0.03 in young (*n* = 35) and adult mice (*n* = 11), respectively; *p*<0.001 and *p*<0.05. Filled red circles, human tumor patients; open red circles, human temporal lobe epilepsy patients. (B) Time constant of recovery from synaptic depression. 144±13 ms in human (*n* = 27, 14 from tumor patients and 13 from epilepsy patients) versus 536±40 ms and 483±91 ms in young (*n* = 35) and adult mice (*n* = 11), respectively; *p*<0.001 in both cases. (C) Time constant of recovery from synaptic depression did not change with age during adulthood. No correlation in the data was found, as indicated by the Pearson's correlation coefficient rho = −0.36, *p* = 0.1. (Data deposited in the Dryad repository, http://doi.org/10.5061/dryad.3723p [56]: Raw data of human and rodent EPSPs in 30 Hz_EPSPs.rar; Numerical data that generated (A–C) in Data S2).

To gain a full quantitative comparison of mouse and human short-term synaptic depression and recovery, we used the mathematical minimal description of activity-dependent short-term synaptic plasticity first proposed by Tsodyks and Markram [17],[25], and extracted best-fit model parameters for each recording (see Materials and Methods). Two out of five fitted parameters were similar between mouse and human synapses (Figure S1A and S1C). Those parameters that differed between human and mouse unitary synapses included a higher “U,” which reflects the probability of synaptic release (Figure 2A; 0.45±0.03 in human versus 0.25±0.02 and 0.29±0.03 in young and adult mice, respectively; *p*<0.001 and *p*<0.05), and the cell membrane time constant, as calculated directly from the experimental traces and by the Tsodyks-Markram model (Figure S1C and S1D). It is relevant to mention that the membrane time constant directly measured from experimental traces and the one calculated by the model differ slightly in definition. The model-driven observable, as extracted by the Tsodyks-Markram model, is obtained by fitting a set of two passive differential equations to the decay of the last EPSP, and for the sole instrumental aim of compensating the passive temporal summation of the successive EPSPs. Its link to the membrane biophysical properties is only indirect, its estimate confidence lower, and it has been included for the purpose of completeness. By far, the largest difference between adult mouse and human synapses was a shorter first-order kinetic time constant, which reflects the recovery from short-term synaptic depression (Figure 2B; 144±13 ms in human versus 536±40 ms and 483±91 ms in young and adult mice, respectively; *p*<0.001). These data indicate that human synapses recover at least three times faster from use-dependent synaptic depression. Similarly fast time constants of recovery have only been reported for facilitating synapses in the ferret prefrontal cortex [26]. Instead, purely depressing synapses in ferret neocortex also have long time constants of recovery of 500 ms up to 900 ms [26], similar to those in mouse and rat neocortex [17],[22]. Furthermore, the time constant of recovery from synaptic depression in adult human synapses (average age 45±11 years) did not change with age during adulthood (Figure 2C; Pearson's correlation coefficient rho = −0.36, *p* = 0.1).

**Figure 3:**
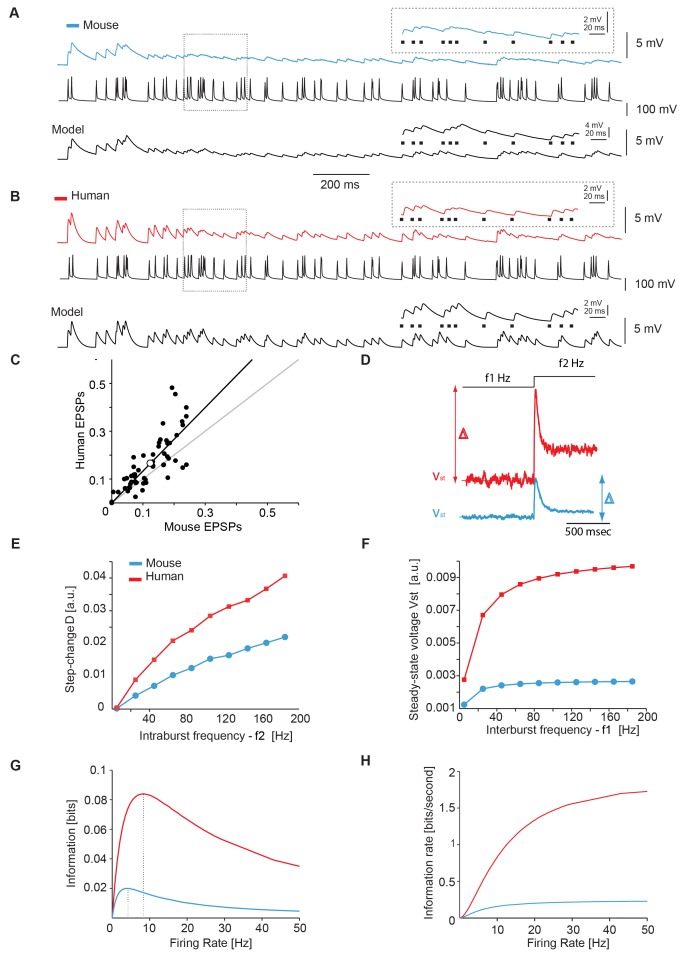
Fast recovering synapses transfer more information. (A,B) Unitary EPSPs in synaptically connected pyramidal neurons of mouse (A, blue) and human (B, red) neocortex generated by a presynaptic Poisson spike train (black). Inset shows enlargement of postsynaptic responses (average of 15 repetitions) to individual presynaptic APs (black dots). Lower traces show model simulations based on the Tsodyks-Markram model [17] with average parameters from experiments in Figure 1. (C) Normalized EPSP amplitudes in human versus mouse pyramidal neurons in response to each corresponding AP in the Poisson spike train in (A) and (B). Gray line has unitary slope, whereas the slope of the fit is 1.33±0.13. Differences are significant with *p*<0.05. Open circle represents the mean and standard deviations (smaller than circle diameter). (D–F) The steady-state membrane potential V_st_ or its transient change Δ was simulated for human and rodent synapses (D), in response to a step increase of the average APs frequency of afferents, active as independent Poisson spike trains. The value of Δ is plotted for increasing values of f_2_ (with f_2_ = 5 Hz) (E), while the value of V_st_ is plotted for increasing values of f_1_ (with f_1_ = f_2_) (F), as predicted by the Tsodyks-Markram model with parameters identified from the experiments. (G,H) Combining the quantal release model of synaptic transmission with the Tsodyks-Markram description, the transfer properties of short-term depressing synapses can be quantified by information theory. Over a broad range of firing frequencies, the mutual information, calculated between peak EPSP amplitudes and presynaptic interspike intervals, reveals a peak at an optimal firing frequency (G); Dividing the mutual information by the firing frequency, the information rate was plotted (H). (Data deposited in the Dryad repository, http://doi.org/10.5061/dryad.3723p [56]: Raw data underlying (A) and (B) in Poisson_Human.rar and Poisson_Mouse.rar; Matlab code that generated (C–H) as well as Figure S2 in Code_Fig3_and_SFig2.rar).

A 3-fold faster recovery from frequency-dependent depression of synaptic connections is likely to affect information transfer between two connected neurons, when repeatedly activated during spike trains [8],[16],[27]. In the neocortex of awake primates, neurons fire irregularly and the instantaneous frequency of each AP varies [28],[29]. We therefore tested whether fast recovery from depression would improve information transfer between two neurons during irregular AP trains with variable firing frequencies (Figure 3). In synaptically connected pairs of pyramidal neurons in mouse neocortex, repeated firing of the presynaptic neuron resulted in a marked reduction of the amplitude resolution by which individual EPSPs could be discerned (Figure 3A). Consequently, some presynaptic APs resulted in very weak postsynaptic voltage changes (Figure 3A and 3C). In contrast, in connected pairs of human cortical pyramidal neurons, all presynaptic APs led to corresponding EPSPs during repeated firing (Figure 3B and 3C), and each EPSP peak amplitude remained well defined during the AP train. Plotting the relative EPSP amplitude at the same AP in the train for mouse and human synapses shows that the peak amplitude of human EPSPs remains better resolved throughout the AP train than mouse EPSPs (Figure 3C). Using the mathematical model and the best-fit parameters, obtained from the short EPSP trains (Figure 2A and 2B), we simulated the response to the irregular synaptic transmission with the exact same AP sequence as applied in the actual recordings: the reduction of synaptic resolution observed in the mouse experiments was replicated (Figure 3A and 3B). With a faster time constant of recovery (144 ms instead of 500 ms), the simulated postsynaptic response resembled the human unitary synaptic responses, whose peak amplitude resolution was maintained throughout the AP train (Figure 3B).

Synapses with faster recovery from depression respond more reliably to presynaptic APs during trains of activity (Figure 3A and 3B), and they may also have a larger dynamic range when signaling abrupt variations in presynaptic firing rate. To investigate this, we tested in model simulations whether fast recovering synapses show larger responses to sudden changes in the frequency of presynaptic AP trains. We simulated 1,500 identical, independent excitatory synaptic afferents impinging on the same postsynaptic neuron, which was modeled as a passive membrane compartment [18],[30]. These virtual synapses were activated asynchronously by independent homogenous point processes to engage short-term synaptic plasticity. Subsequently the average activation frequency was step-changed as in a burst, to test how well synapses would detect and respond to phasic presynaptic activity [30]. Synapses with fast recovery from depression indeed conferred a higher dynamic range of synaptic transmission, as well as an increased sensitivity to small changes in presynaptic network activity time course (Figure 3D–3F). As we swept through different intra- (Figure 3E) and inter-burst frequencies (Figure 3F), the faster recovery from depression always provided the postsynaptic neurons with a larger sensitivity to their synaptic inputs. These results indicate that synapses that recover faster from depression, as we observed in human neocortical synapses, are equipped to relay fine variations in the instantaneous firing frequency, more reliably than synapses that slowly recover.

As synapses that recover quickly from depression operate with an increased bandwidth during repeated activation, they may be able to relay more information than synapses that slowly recover from depression. To test this, we numerically calculated the mutual information between the amplitude of the postsynaptic membrane potentials and the length of the inter-spike intervals of a train of corresponding presynaptic APs. Using the mathematical model and the best-fit parameters (Figure 2), the Shannon's formalism of information theory [31] applied to depressing synapses [7] provided a quantitative measure for the information transfer through a synapse (see Materials and Methods). We found that synapses that recover quickly from depression convey approximately four times more information at peak levels (Figure 3G). The average presynaptic firing frequency, corresponding to the optimal information transfer [7], was higher in quickly recovering synapses (9.1 Hz) compared with slowly recovering synapses (4.5 Hz). Quickly recovering synapses were consequently able to sustain larger information transfer rates at higher firing frequencies (Figure 3H), and information transfer rate saturated less prominently at higher frequencies than for slowly recovering synapses. These findings suggest that human neocortical depressing synapses that show fast recovery from depression may relay more information than depressing neocortical synapses found in the mouse brain.

**Figure 4:**
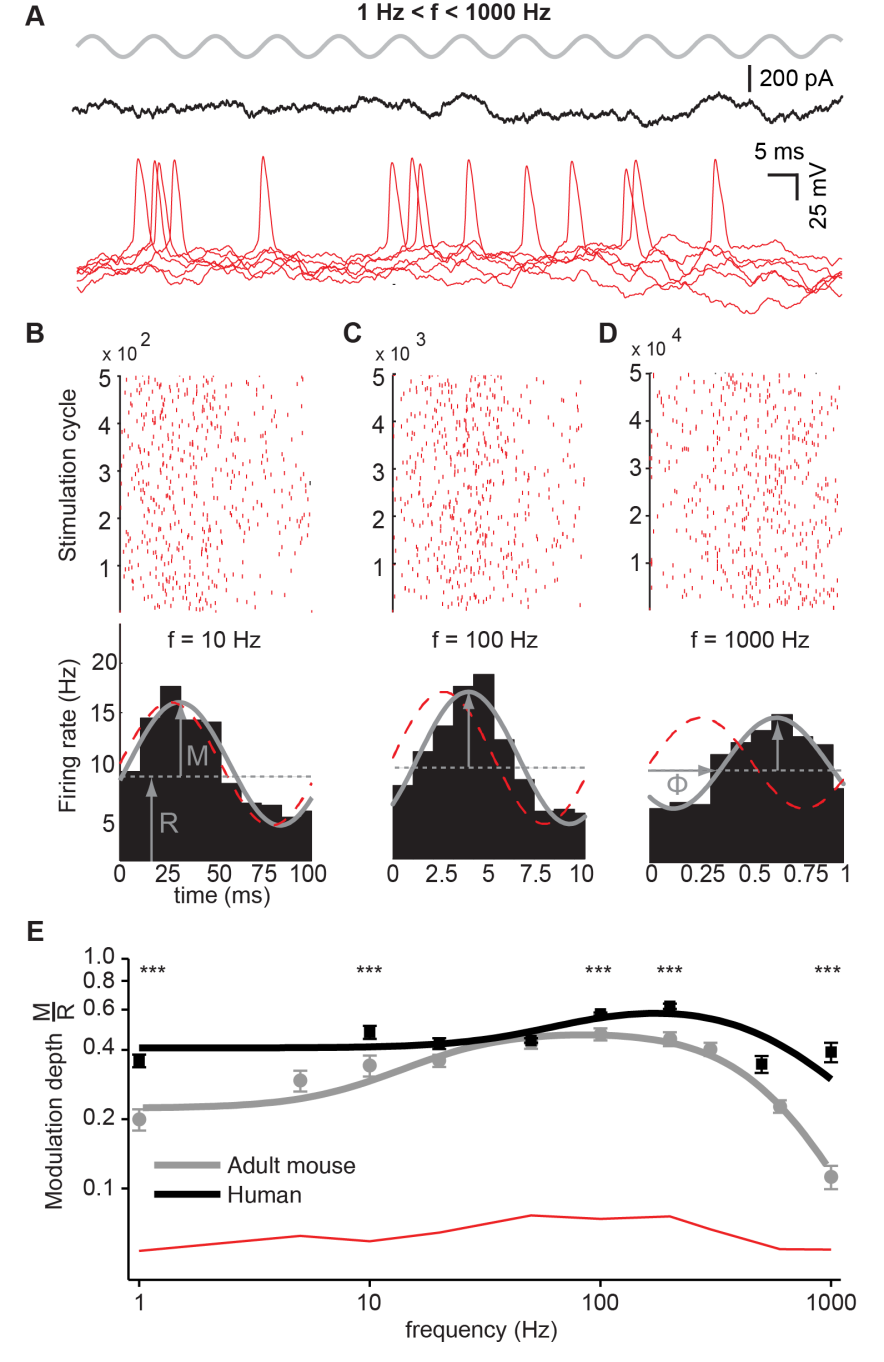
Human pyramidal neurons track input signals beyond 500 cycles/s. (A) Top trace (gray): weak amplitude sinusoidal currents, injected with distinct oscillation periods (1–1,000 cycles/s). Middle trace (black): injected randomly fluctuating additional current component. Lower trace: AP firing by human pyramidal neuron in response to the total injected current. (B–D) Across successive cycles and for distinct sine input oscillations (10, 100, 1,000 cycles/s), APs fired by a human pyramidal neuron are displayed in raster diagrams (B–D, upper panels) and are quantified by a peristimulus time histogram (PSTH, lower panels), which estimates the instantaneous firing probability. (E) Modulation depth (i.e., peak modulation magnitude over the mean rate, M/R) as a function of Fourier frequency for human (*n* = 13, black) and mouse cells (*n* = 14, gray). Larger frequencies imply faster input oscillations. Markers indicate mean ± SEM, while the thick solid traces are fits obtained with a rational complex function (see the Materials and Methods and Methods S1). The red line indicates the significance level for the data, obtained by computing the modulation depth over surrogate data, obtained shuffling the interspike intervals. (Data deposited in the Dryad repository, http://doi.org/10.5061/dryad.3723p [56]: Raw data underlying A–E and Figure S3 in Data_Fig4_and_SFig3_Human.rar and Data_Fig4_and_SFig3_Mouse_Part1 to Part4.rar; scripts used for analysis and that generated (A–E) are also included in Data S4.xlsx).

Adult human neocortical neurons receive thousands of excitatory synapses, with estimates for adult layer 2/3 pyramidal neurons as high as 30,000, about twice as many as rodent layer 2/3 pyramidal neurons [32]. When each of these synapses operates with high resolution at high bandwidth and maintains reliability during bursts of activity, as our findings suggest, the question arises whether human pyramidal neurons can actually encode fast-varying temporal inputs in the AP train. To test whether human pyramidal neurons can precisely time their AP firing to rapidly changing inputs, we measured the temporal modulation of the neuronal output firing probability of human pyramidal neurons, during somatic injection of sinusoidal currents in whole-cell recordings (Figure 4) [33]–[35]. Neurons simultaneously received an additional, randomly fluctuating, current component (Figure 4A; see Material and Methods) that induced an irregular firing regime with low average rate (13.3±1.6 Hz, CV_ISI_ = 1.06±0.02, *n* = 13 human and 11.9±0.6 Hz, CV_ISI_ = 0.8±0.02, *n* = 14 adult mouse neurons). While the fluctuating component *per se* resulted in a uniform probability of AP firing in time, the superimposed weak amplitude small sinusoidal currents modulated in time the instantaneous firing probability, with the same period of the input (Figure 4B–4D). Under these conditions, the timing of AP firing in human neurons was more strongly modulated both by large and small input periods, going up to 1,000 cycles/s (Figure 4E), indicating that human neurons could encode finer and rapidly changing temporal features of their input into AP timing.

The data in Figure 4E represent typical band-pass behavior for both human (*n* = 13) and murine cells (*n* = 14), where the continuous black and grey lines represent an equivalent passive analogue electronic filter. The pass band of human neurons was shifted to higher Fourier frequencies (low frequency “pole” and “zero” cut-off located at 52 and 82 Hz, respectively, compared to 9 and 20 Hz for mouse cells) showing a higher level of selectivity (high frequency “pole” cut-off located at 524 and 565 Hz in humans and rodents, respectively). Additionally, the multiplicity but not the location of the high Fourier frequency pole differed between human and mouse cells: a value of 2 for the latter group implies that the negative slope of the Fourier transfer function, above a cut-off frequency of ∼500 Hz, is larger in mouse than in human neurons. This transfer function for adult temporal cortex mouse neurons is consistent with what was previously found for layer 5 pyramidal cells of the primary somatosensory cortex of juvenile rats [31]. Taken together, these results suggest that human neurons are more selectively tuned for high Fourier frequency components of their inputs and that their attenuation while relaying very fast signals, with Fourier components beyond 500 cycles/s, decays significantly less rapidly than in adult mouse cells.

**Figure 5:**
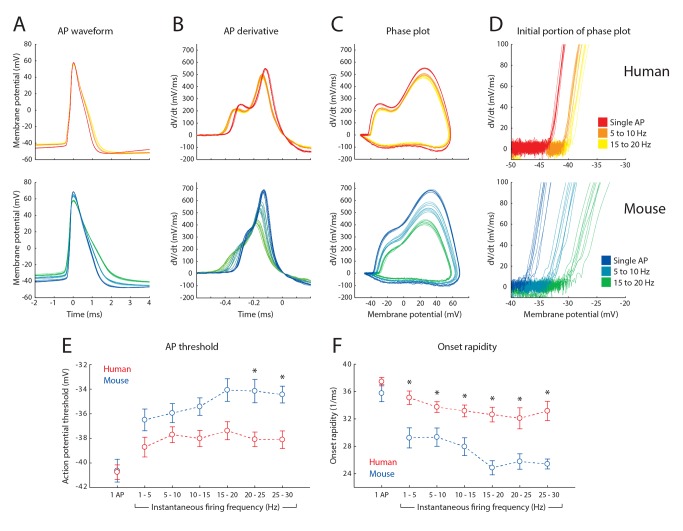
Fast AP onsets are maintained during repeated firing in human pyramidal neurons. (A–D) Waveforms and derivatives of APs recorded from a human (top traces) and mouse pyramidal neuron (bottom traces). Different colors correspond to APs with different instantaneous firing frequencies (see inset, (D)). (A) AP waveform in time (ms) aligned to timing of the AP peak. (B) First derivative of rising phase of AP, aligned to timing of AP peak. (C) Phase plot of APs with the AP derivative dV/dt versus membrane potential (mV). (D) Magnification of initial portion of phase plot shown in (C). (E) Summary data of average AP threshold of human (red; *n* = 23) and mouse (blue; *n* = 12) neurons, displayed versus firing frequency. Data are presented as means ± SEM for single APs, and APs fired in trains binned in 5 Hz bins according to instantaneous firing frequency. Asterisks indicate *p*<0.005. (F) Same as (E), for average AP onset rapidity. (Data deposited in the Dryad repository, http://doi.org/10.5061/dryad.3723p [56]: Raw data underlying (A–F) as well as Figure S4 in Data_Fig5_and_SFig4.rar; Numerical data that generated (E) and (F) as well as Figure S4 in Data S5.xlsx).

From theoretical considerations it was predicted that tracking of fast input frequencies by neurons depends on the rate of onset of APs [35],[36]. We tested whether human neocortical pyramidal neurons have substantially faster AP onset kinetics than mouse pyramidal neurons. Single APs of human (26–47 years, *n* = 23 neurons) and adult mouse (10–11 weeks, *n* = 12 neurons) temporal cortex pyramidal neurons had similar waveform and duration, but different kinetic features (Figure 5A). However, APs fired in trains with varying instantaneous frequencies showed strong differences between human and mouse pyramidal neurons. In particular, the rising phase of APs fired by mouse neurons slowed down more with increasing firing frequency than APs generated by human neurons (Figures 5B, S4E, and S4F; *p*<0.005). At higher firing frequencies the threshold for AP generation was elevated in mouse pyramidal neurons compared to human neurons (Figure 5E). Importantly, at instantaneous firing frequencies of 1 to 30 Hz, mouse APs had reduced onset rapidity compared to APs fired by human pyramidal neurons (Figure 5D and 5F, *p*<0.005 for all frequencies). A recent study reported that in order for neurons to track fast varying inputs, with Fourier components up to 1,000 cycles/s, the AP onset rapidity needed to be above 30 mV/ms per mV [35]. APs fired by human pyramidal neurons had mean onset rapidity values above 32 mV/ms per mV for all firing frequencies tested (Figure 5E). These results show that APs generated by human neurons have a sufficiently fast onset to account for the ability of these neurons to track very fast inputs, with Fourier components up to 1,000 cycles/s.

Our findings show that synaptic communication between human neocortical pyramidal neurons has higher bandwidth due to fast recovery from depression and that these neurons are equipped to track fast input Fourier components and encode these into timing of their spikes. Transfer of information between neurons through chemical synaptic transmission is elementary to cognition, and processes of short-term plasticity at these synapses encode information [16]. Studies on rodent excitatory cortical synapses show that short-term facilitation of synaptic strength may optimize information transfer in particular during spike bursts [27],[37]. Based on findings in the rodent brain, it is assumed that purely depressing synapses may be better suited to transmit information for single spikes or short bursts rather than for trains of APs [16]. In contrast to these observations, we show here that purely depressing synapses in the human brain can actually transfer substantial amounts of information during spike trains, because recovery from depression is fast. We find that information transfer at depressing synapses with fast recovery is optimal at alpha band frequencies (8–12 Hz), and information transfer rate increases well into the beta and gamma band frequency range, suggesting that these synapses can be involved in active cortical computation, during cognition. This may unveil a fundamental difference with purely depressing synapses that slowly recover from depression in the neocortex of mice and other laboratory animals, which we find to have optimal frequencies of information transfer in the lower theta band range with no increase in information transfer rate at higher frequencies. These synapses may be better suited for a different range of cortical processes [38].

In our study, we did not include polysynaptic events that have been described previously [39], and we restricted our attention to monosynaptic connections between pyramidal cells from L2/3 in the anterior medial temporal cortex, where polysynaptic events did not seem to play a prominent role. In rodent synapses, the amount and speed of short-term synaptic depression and its recovery are dependent on temperature as well as divalent ion concentrations [40],[41]. It is at present unknown to what extent synaptic depression and recovery in human synapses depend on temperature and divalent ion concentrations. Combined with a lack of information on the actual calcium and magnesium concentrations and temperature at synapses in the brain of awake behaving mice and humans, it is not feasible to predict what the speed of recovery from depression in mouse and human cortical synapses will be in the intact brain during behavior. Nevertheless, we show here that under defined experimental conditions in which temperature and extracellular divalent ion concentrations are controlled, human and mouse temporal cortex synapses show marked differences in the speed of recovery from synaptic depression. This suggests that differences in protein complexity of synaptic protein networks between mouse and human synapses [10] may translate into different functional properties of short-term synaptic plasticity.

Postsynaptically, the outcome of short-term synaptic plasticity processes is translated into AP firing to relay information [13]. The brain not only keeps track of the number of spikes occurring in large windows of time, but spike timing can have meaning down to millisecond precision [42]. Spike timing with a temporal resolution smaller than the time scales of sensory and motor signals, even at sub-millisecond levels, can encode significant amounts of visual information [42]. Cortical pyramidal neurons time their AP firing in relation to the timing of synaptic input [43],[44]. However, populations of rodent pyramidal neurons fail to time their spiking based on rapidly varying inputs components that change faster than 200–300 cycles/s. This may suggest that, during ongoing synaptic membrane potential fluctuations, rodent neurons do not regularly encode and transmit downstream information with sub-millisecond precision. We find that populations of human pyramidal neurons can regularly time AP firing with sub-millisecond precision and that these APs maintain rapid onset kinetics, which can account for such precision of spike timing. Rapid onset kinetics of somatic APs are predicted by Hodgkin-Huxley-based models for AP generation when the spatially extended morphology of neurons and AP propagation from the axon initial segment to the soma are taken into account [45]. The observed fast onset rapidity of APs in adult human neurons can indeed partly be explained by human pyramidal neuron morphology [46]. In particular, the electrical load imposed by the large dendritic tree of adult human layer 2/3 pyramidal neurons compared to adult rodent pyramidal neurons on the axon initial segment induces a larger onset rapidity of the AP and higher frequency tracking capabilities. In conclusion, our data show that elementary circuits in the human neocortex of connected pyramidal neurons that underlie cognition can operate at a substantially higher bandwidth and temporal resolution for information encoding than rodent neurons in response to the high information content synaptic transmission they receive.

## Materials and Methods

### Human Neocortical Slice Preparation

All procedures on human tissue were performed with the approval of the Medical Ethical Committee of the VU University Medical Center and in accordance with Dutch licence procedures and the Declaration of Helsinki. Human slices were cut from anterior medial temporal cortex that had to be removed for the surgical treatment of deeper brain structures for epilepsy or tumors with written informed consent of the patients (aged 18–61 years) prior to surgery. Anaesthesia was induced with intravenous fentanyl 1–3 µg/kg and a bolus dose of propofol (2–10 mg/kg) and was maintained with remyfentanyl 250 µg/kg/min and propofol 4–12 mg/kg. Immediately following removal from the brain, neuropathologists assessed whether it was normal or diseased tissue and only those samples that were designated as normal were used in the present study.

After resection, the neocortical tissue was placed within 30 seconds in ice-cold artificial cerebrospinal fluid (aCSF) slicing solution which contained in (mM): 110 choline chloride, 26 NaHCO_3_, 10 D-glucose, 11.6 sodium ascorbate, 7 MgCl_2_, 3.1 sodium pyruvate, 2.5 KCl, 1.25 NaH_2_PO_4_, and 0.5 CaCl_2_ (300 mOsm) [23],[24],[45] and transported to the neurophysiology laboratory, which is located within 500 m from the operating room. The transition time between resection of the tissue and the start of preparing slices was less than 15 minutes.

Neocortical slices (350–400 µm thickness) were prepared in ice-cold slicing solution, and were then transferred to holding chambers in which they were stored for 30 minutes at 34°C and for 30 minutes at room temperature before recording in aCSF, which contained (in mM): 126 NaCl; 3 KCl; 1 NaH_2_PO_4_; 1 MgSO_4_; 2 CaCl_2_; 26 NaHCO_3_; 10 glucose (300 mOsm), bubbled with carbogen gas (95% O_2_/5% CO_2_).

### Mouse Neocortical Slice Preparation

All procedures were approved by the VU University's Animal Experimentation Ethics Committee and by the Ethics Committee of the Department of Biomedical Sciences of the University of Antwerp. C57Bl6 mice (2–11 weeks of age) were decapitated prior to slice preparation, in accordance with Dutch and Belgian licensed procedures. Brains were rapidly removed and dissected using the same solutions for slicing and storage as used in preparation of human brain slices. Coronal slices (300–450 µm thickness) were cut from the prelimbic region of the medial prefrontal cortex (mPFC) (P12–36) or the temporal association cortex (TC) (8–11 weeks). As during preparation of human brain slices, slices were allowed to recover for 30 minutes at 34°C followed by 30 minutes at room temperature in the same solution used for recording. Slices of adult mice were allowed to recover for 15 minutes at 34°C in the same solution used for slicing and then transferred to a chamber containing the same solution used for recording, at room temperature.

### Electrophysiology

Neocortical slices were visualized using either infrared differential interference contrast (IR-DIC) microscopy or Hoffman modulation contrast. After the whole cell configuration was established, membrane potential responses to steps of current injection were used to classify each cell electrophysiologically. Cells were loaded with biocytin through the recording pipette for post hoc identification. All experiments were performed at 32°C–35°C. None of the neurons recorded from showed spontaneous epileptic-form spiking activity. All experiments were performed in the absence of blockers of GABAergic synaptic transmission.

Recordings were made using Multiclamp 700A/B amplifiers (Axon Instruments) sampling at intervals of 4 to 100 µs, and low-pass filtered at 10 to 30 kHz. Recordings were digitized by pClamp software (Axon), by LCG software [47], or custom written scripts in Igor Pro, and later analyzed off-line using custom written Matlab scripts (The Mathworks). Patch pipettes (3–5 MOhms) were pulled from standard-wall borosilicate capillaries and filled with intracellular solution containing (in mM): 110 K-gluconate; 10 KCl; 10 HEPES; 10 K-phosphocreatine; 4 ATP-Mg; 0.4 GTP, pH adjusted to 7.2–7.3 with KOH; 285–290 mOsm, 0.5 mg/ml biocytin.

Post hoc visualization and neuron identification using biocytin labelling was performed as described previously [48],[49]. Pyramidal neurons were classified based on morphological and electrophysiological criteria. Input resistances were calculated from the steady state response to hyperpolarizing current pulses (mean ± SEM): human R_in_ = 70±6 MΩ (*n* = 27), young mouse R_in_ = 84±3 MΩ (*n* = 45), adult mouse R_in_ = 102±7 MΩ (*n* = 26) (adult mice significantly different from human, *p*<0.01). Resting membrane potentials (not corrected for liquid junction potentials – mean ± SEM): human V_rest_ = −73.2±0.8 mV (*n* = 27), young mouse V_rest_ = −69.6±0.7 mV (*n* = 45), adult mouse V_rest_ = −74.5±1.1 mV (*n* = 26) (adult mice and human significantly different from young mice (*p*<0.02), but not different amongst each other). These numbers were taken into account when injecting current to test whether human pyramidal neurons can time their AP firing to high frequency inputs. The baseline current injected was set to keep iso-frequency firing close to 10–15 Hz.

### Frequency-Dependent Short-Term Synaptic Depression (STD)

The model of Tsodyks and Markram [17],[22],[25] was employed to quantitatively characterize use-dependent short-term depression of EPSPs amplitude in response to defined trains of presynaptic APs. This description refers to the existence of generic resources for neurotransmission, without distinguishing between presynaptic (e.g., the ready-releasable pool of vesicles) and postsynaptic biophysical components (e.g., desensitization of AMPA receptors). The model is identified by five numerical parameters [50]: A, the absolute synaptic efficacy; U, the fraction of resources consumed by each AP; 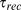, the time constant of recovery from exhaustion of available resources; 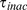, the synapses' time constant to transit between active and inactive states; 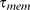, the membrane time constant, as defined in a leaky integrate-and-fire model. The peak amplitude of the *n*th postsynaptic response, indicated by E_n_, is given by E_n_ = (A U R_n_), where the dynamical variable R is the running value of the available resources. Indicating the times of successive APs, as 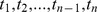, the model responses 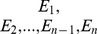 are obtained by 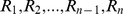 upon numerical iteration: 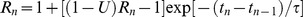. The same numerical method was employed for both simulating model responses, as well as to search for parameters {A, U, τ} that optimally reproduce the experimental data after least-square fitting.

### Novelty-Detection in Presynaptic Firing Rate by a Population of Synaptic Afferents

A passive R-C circuit was mathematically defined to mimic temporal integration of postsynaptic responses in a point-neuron with membrane time-constant of 10 msec. Then, 1,500 identical synaptic afferents impinging on this neuron were activated, each by an independent realization of an identical Poisson point-process. The mean frequency of this random process was step-changed from f_1_ to f_2_, after several seconds of simulation lifetime. Each individual model synapse relayed the occurrence of a presynaptic AP in a use-dependent manner, according to the Tsodyks-Markram model described above. Without losing any generality, simulated postsynaptic responses were expressed and plotted in arbitrary units, normalizing voltage responses to the product of the (unspecified) neuronal input resistance and maximal synaptic efficacy A.

### Quantifying Temporal Information Transfer at a Single Synapse

As an alternative to the Tsodyks-Markram model, we considered its non-deterministic formulation, which combines the classical quantal model [51],[52] with use-dependent short-term depression as in Fuhrmann and colleagues [7]. We considered *n* = 5 release sites [24] and the average quantal content A/N, with A being the maximal synaptic efficacy of the deterministic Tsodyks-Markram model. The last choice implies that on the average of many repeated trials, the non-deterministic model responses quantitatively correspond to the predictions of the Tsodyks-Markram deterministic formulation. The coefficient of variation of the quantal content was set to 0.4, choosing its standard deviation as 0.4 A/N. The coefficient of variation's value was taken from an example in the literature [7] and its numerical value scales proportionally the mutual information calculations and thus does not affect our conclusions. To demonstrate the previous statement we explored different values of CV of the simulated quantal content (i.e., 0.2, 0.4, 0.6, 0.8) (see Figure S2). The parameter has, therefore, no qualitative effect, but only a scaling effect. As opposed to a classic quantal model, the probability of release is non-stationary, and it is computed as the product between the fixed probability that a release site contains a vesicle (U) and the probability P_v_(t) that a vesicle is available at a given time t. In the lack of any presynaptic AP, P_v_(t) recovers exponentially to 1 with a recovery time-constant τ, while immediately after an AP this probability is decreased by a proportional amount, 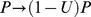. This model allows one to apply information theoretical methods [15], extended to probabilistic synaptic transmission, and lead to quantify mutual information between the set of postsynaptic responses to a train of presynaptic spikes, and the corresponding set of interspike intervals [7]. The last are assumed to act as a source of (arbitrary) temporal information.

Several average presynaptic firing frequencies were considered (0.01–100 Hz). For each average frequency, a realization of a Poisson point-process was generated to simulate the time of occurrence of 10,000 presynaptic spikes fired at such an average frequency. The marginal probability density of the postsynaptic amplitudes was estimated, and the corresponding conditional probability density, given the instantaneous probability of release, derived under the same assumptions of Fuhrmann and colleagues [7]. Conditional entropies were computed according to the definition of Shannon [31], and mutual information computed as their difference.

### Modulation of Firing by Noise + Sine Injection and Fit of the Transfer Function

To evaluate the dynamical transfer properties of neuronal discharge, in response to rapidly varying inputs, a sinusoid of amplitude *I_1_* and frequency F (1–1,000 cycles/s) was applied simultaneously to a DC baseline *I_0_* and to a randomly fluctuating waveform, under current-clamp stimulation:
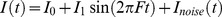
(1)The fluctuating component *I_noise_(t)* was synthesized as an exponentially filtered Gaussian white-noise realization, mimicking at the soma the consequences of a barrage of balanced background excitatory and inhibitory irregular synaptic inputs, as described previously [26],[31],[32],[53]. *I_noise_(t)* had zero-mean, variance *s^2^* and correlation length *τ_I_* = 5 msec, and was generated by means of the LCG software [47] iterating the following expression at the same rate of the sampling interval (i.e., 1/dt = 20 kHz),
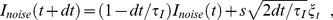
(2)where {*ξ_t_*} is a sequence of independent pseudo-random numbers with normal distribution [54]. Depending on the cell input resistance and rheobase, the current DC baseline *I_0_* and the random fluctuation amplitude *s* were adjusted so that for *I_1_* = 0 pA, neurons responded (i) with low mean rate (∼10–15 Hz), (ii) highly irregular inter-spike intervals, and (iii) subthreshold voltage random fluctuations (5–10 mV) as observed in cortical recordings *in vivo* [53]. Finally, *I_1_* was chosen as a fraction of *I_0_* (e.g., *I_1_* = 50 pA, *I_1_* = 400 pA) and each stimulation *I(t)* lasted 50 s and was followed by a long recovery time of at least 50 s.

Raw voltage traces were recorded for different values of F and offline processed in MATLAB (The Mathworks). Individual spike times {t_k_}, k = 1,2,3,…, occurring across subsequent input oscillation cycles, were extracted by a peak-detection algorithm and then normalized to the corresponding oscillation period: i.e., t_k_→(t_k_ % F^−1^), where % indicates the remainder of integer division. Peristimulus time histograms (PSTH) with 30 bins, were then computed and normalized to represent the instantaneous discharge rate. Three free parameters *r_0_*, *r_1_*, and φ of the sinusoidal function
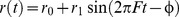
(3)were optimized to best-fit in the least-squares sense each PSTH by r(t), through the Levenberg-Marquardt algorithm [54]. The same procedure was repeated on surrogate spike-train data, obtained randomly shuffling the interspike-intervals {(t_k+1_−t_k_)} to obtain the minimal level of significance for the estimates of *r_1_* and φ.

To fit experimentally measured amplitude and phase response data, we used a linear model, as described in [33], reminiscent of a passive analogue electronic filter. Briefly, the modulation depth *r_1_(f)/r_0_* and the phase 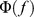 were taken as the magnitude and phase of the impulse response of a linear dynamical system described, in the Fourier domain, by the following rational complex function:
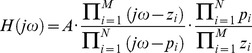
(4)where the polynomials roots 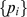 and 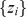 are known as “poles” and “zeros” cut-off of the transfer function, respectively, and *A* is the low-frequency gain. The transfer function in Equation 4 was used to fit the magnitude and phase responses over the population of cells, as shown in Figure 4E, and the fit was weighted by the inverse of the standard deviation of each data point. The two datasets, corresponding to human and adult mouse cells, were fit with functions containing a single zero (i.e., M = 1), and either two or three poles (i.e., N = 2–3). In both cases, N>M accounts for the power-law decay of the magnitude of the transfer function at frequencies above the cut-off frequency according to the relation
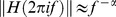
(5)With 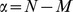. To model the fact that the phase response does not saturate for high Fourier frequencies at integer multiples of 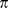 [34], we included in our formulation a constant propagation delay 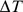, which takes into account, among other things, the time it takes for the spike to travel from its originating zone to the point at the soma where it is recorded (see [33],[34] for a discussion).

### Analysis of Action Potential Waveform

In experiments aimed at examining AP-waveform (Figure 5), data were acquired with 4 or 10 µs sampling intervals, low-pass filtered at 30 kHz, and filtered offline at 15 kHz. Bridge balance was adjusted manually and pipette capacitance was compensated for. Recordings were excluded if bridge balance exceeded 12 MOhm. For analysis, all APs with instantaneous firing frequencies up to 30 Hz were pooled and binned for all recordings from a cell. Traces with resting membrane potentials above −60 mV and APs where the linear fit to obtain onset rapidity had R∧2 values <0.95 were excluded from analysis. The various AP parameters were calculated for each AP in a train, as follows: the AP threshold was defined as the membrane potential at the point that the velocity of the AP exceeded 10 mV/ms [55]. The AP peak voltage was determined as the absolute membrane potential measured at the peak of the AP waveform. The AP amplitude was calculated as the difference in membrane potential between the AP threshold and the AP peak voltage. Maximum rate of rise was defined as the maximum dV/dt value reached during the AP (calculated between adjacent points). Onset rapidity was defined as the slope of a linear fit to the AP phase plot (dV/dt versus V, with unit 1/ms) at dV/dt = 30 mV/ms.

The first AP in a train was considered a single AP. For all APs that followed, the instantaneous AP firing frequency was calculated as: 1/(time since previous spike). For subsequent analysis, APs with instantaneous firing frequencies up to 30 Hz were binned in frequency bins of 5 Hz. For each neuron, the mean value of a given AP parameter in a frequency bin was then obtained by averaging over all APs falling in that frequency bin. AP amplitude adaptation was calculated by dividing the mean amplitude of APs in a frequency bin by the mean amplitude of single APs. Maximum rate of rise adaptation was calculated by dividing the mean maximum rate of rise of APs in a frequency bin by the mean maximum rate of rise of single APs. Threshold variance was calculated as the standard deviation of AP thresholds for all APs within a frequency bin. Differences in AP features between human and mouse neurons were tested for significance using independent samples t-tests, with a Bonferroni corrected *p*-value to account for family-wise error rate.

### Connectivity and Connection Probability

The surgeon obtained tissue samples from human temporal cortex in variable forms, in a patient-dependent manner. We could reliably determine the location of the pia and the white matter to adjust the slice angle to maintain the apical dendritic tree within the slice, but had less control of the slicing orientation on the coronal/sagittal axis and relative to individual gyri (unlike in mouse brain, where landmarks such as midline or corpus callosum help in positioning the sample). Given these factors, we have not conducted a systematic analysis of connection probability between mouse and man to provide reliable estimates and comparisons of synaptic connectivity ratios between species. Rather, we focused only on finding direct monosynaptic connections for investigation and subsequent analysis.

Data discussed in this paper has been deposited in the Dryad repository: http://doi.org/10.5061/dryad.3723p [56].

## Supporting Information

Figure S1 **Tsodyks-Markram model parameter comparisons for mouse and human synapses.** (A) Absolute synaptic efficacy, proportional to the amplitude of the first evoked EPSP. (mean ± SEM) 3.7±0.5 mV human, 5.6±1.1 mV for young mouse synapses and 1.6±0.4 mV for adult mouse synapses, *p*>0.05; human *n* = 27 (14 from tumor patients and 13 from epilepsy patients, see Materials and Methods; young mouse *n* = 35; adult mouse *n* = 11). (B) Inactivation time constant. (mean ± SEM) 2.2±0.3 ms human, 1.9±0.1 ms for young mouse synapses and 2.1±0.3 ms for adult mouse synapses, *p*>0.05; human *n* = 27 (14 from tumor patients and 13 from epilepsy patients, see Materials and Methods; young mouse *n* = 35; adult mouse *n* = 11). (C) Membrane time constant measured from membrane potential deflection upon short current pulse injection (mean ± SEM) 19.5±1.0 ms human, 25.1±0.4 ms for young mouse synapses and 24.6±2.0 ms for adult mouse synapses, adult and young mice have significantly higher membrane time constants than humans (*p*<0.05); human *n* = 27; young mouse *n* = 27; adult mouse *n* = 15. (D) Membrane time constant, as in a leaky integrate-and-fire neuron model, (mean ± SEM) 28±3 ms human, 22±1 ms for young mouse synapses and 42±4 ms for adult mouse synapses, adult mice have significantly higher membrane time constants than humans and young mice (*p*<0.05 and *p*<0.001, respectively); human *n* = 27 (14 from tumor patients and 13 from epilepsy patients, see Materials and Methods; young mouse *n* = 35; adult mouse *n* = 11). (E) More examples of the time course of synaptic depression on a 30 Hz train of EPSPs. 8 pulses+1 recovery pulse 500 ms after the 8th pulse for mouse connections and 300 ms after the 8th pulse for a human connection (of the *n* = 27 pairs measured in human slices, six were probed with 500 ms and 21 with 300 ms between the 8th and 9th pulse).
(TIF)

Figure S2 **Different values of CV of the simulated quantal content (i.e., 0.2, 0.4, 0.6, 0.8) only scales the mutual information.** From (E), we can conclude that in the model the CV parameter has no qualitative effect. Changing its value results in a scaling effect only. The figure was obtained with a smaller number of simulated Poisson spikes, it therefore appears noisier than Figure 3G and 3H.
(TIF)

Figure S3 **When the dynamical response properties of human (black) and rodent (gray) pyramidal neurons are analyzed in terms of phase Φ(** ***f*** **), instead of response magnitude (** **Figure 4** **), very similar profiles in human and mouse neurons are observed across the Fourier frequencies.** Mouse neurons revealed a more prominent low Fourier frequency phase advance than human neurons, as a direct consequence of more prominent spike-frequency adaptation.
(TIF)

Figure S4 **Action potential parameter comparisons for mouse and human neurons.** (A–F) Quantification of various AP features of human (red) and mouse (blue) neurons, displayed versus firing frequency. Data are presented as means ± SEM. for single APs, and APs fired in trains binned in 5 Hz bins according to instantaneous firing frequency. Asterisks indicate *p*<0.005. (A) AP amplitude, in mV from threshold. (B) AP peak voltage. (C) AP amplitude adaptation. (D) Threshold variability, presented as the standard deviation of AP thresholds. (E) Maximum rate of rise. (F) Maximum rate of rise adaptation. For more details on how AP features were calculated, see Methods.
(TIF)

Figure S5 **Average 10 Hz 10-spike long Poisson train is depicted, together with resulting synapse raw responses, for the deterministic Tsodyks-Markram model.**
(TIF)

Figure S6 **Increasing (average) presynaptic spiking rate, the postsynaptic current (PSC) histogram shifts to weaker peak values (i.e., short-term depression).**
(TIF)

Table S1 **Parameters derived by the Tsodyks-Markram model for dynamically depressing synapses.**
(DOCX)

Table S2 **Kinetic parameters from EPSPs.**
(DOCX)

Table S3 **Intrinsic cell properties, calculated from resting membrane potential and steady-state response to hyperpolarizing current injection pulse.**
(DOCX)

Data S1 **Numerical data used to generate** **Figure 1B–E** **and Figure S1A–D.** Also shown in files ‘Figure 1A.rar’ and ‘30Hz_EPSPs.rar’ in Dryad repository http://doi.org/10.5061/dryad.3723p [56].
(XLSX)

Data S2 **Numerical data used to generate** **Figure 2A–C** **.** Also shown in file ‘30Hz_EPSPs.rar’ in Dryad repository http://doi.org/10.5061/dryad.3723p [56].
(XLSX)

Data S3 **Numerical data used to generate Figures 5 and S4** **.** Also shown in Dryad repository http://doi.org/10.5061/dryad.3723p [56].
(XLS)

Index S1 **Index file (index S1.xlsx): List and content description of all files and folders present in the Dryad repository (doi:10.5061/dryad.3723p).**
(XLSX)

Methods S1 **Extension of the information theory mathematical framework to dynamic synapses.**
(DOCX)

## Abbreviations

AP: action potential
EPSP: excitatory postsynaptic potential
SEM: standard error of the mean
