# Supplementary figures and images for: High Bandwidth Synaptic Communication and Frequency Tracking in Human Neocortex
Source: PLoS Biol. 2014 Nov 25;12(11):e1002007. doi: 10.1371/journal.pbio.1002007 (PMC4244038; doi:10.1371/journal.pbio.1002007)

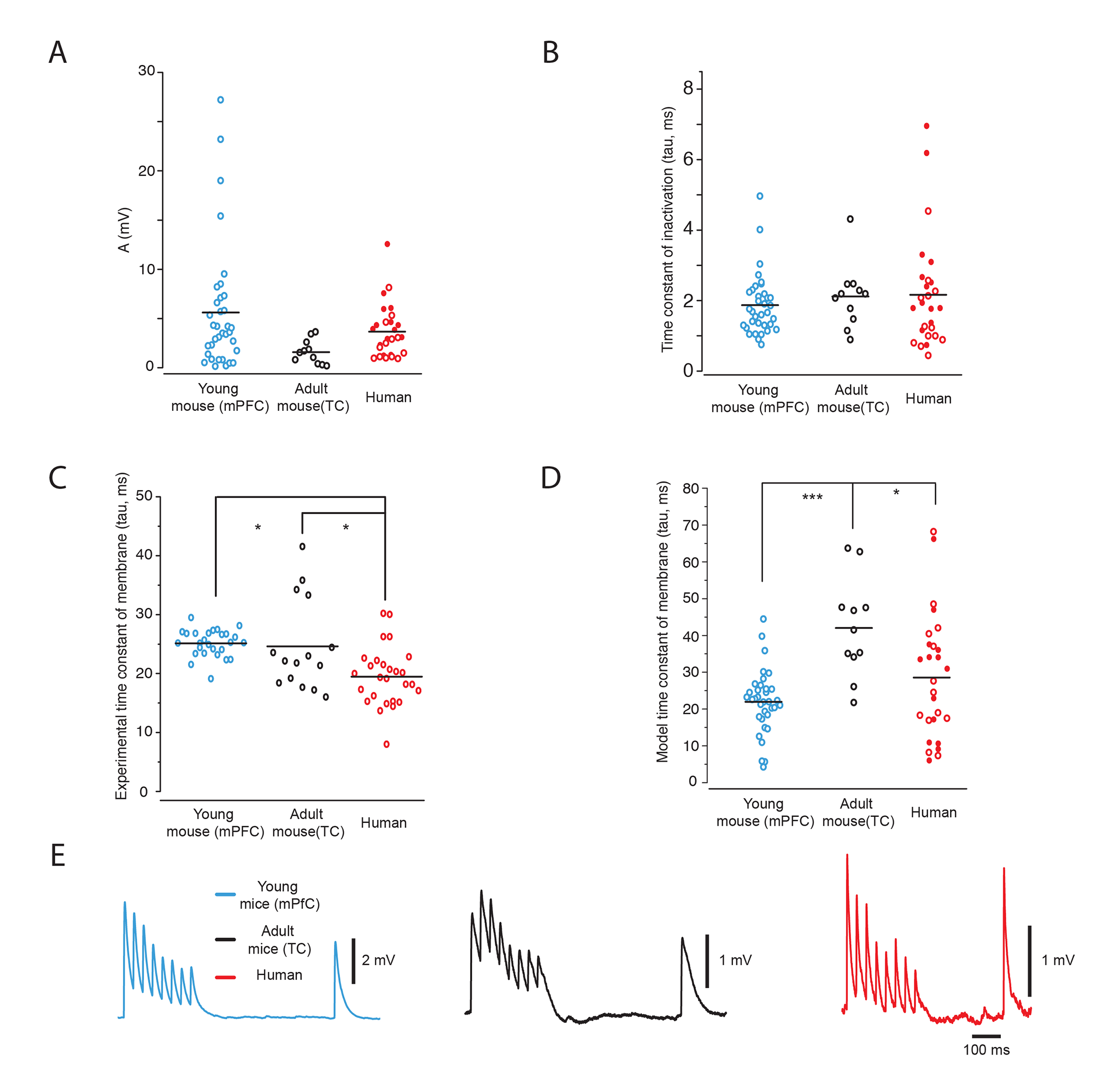

Supplement: Figure S1 — Tsodyks-Markram model parameter comparisons for mouse and human synapses. (A) Absolute synaptic efficacy, proportional to the amplitude of the first evoked EPSP. (mean ± SEM) 3.7±0.5 mV human, 5.6±1.1 mV for young mouse synapses and 1.6±0.4 mV for adult mouse synapses, p>0.05; human n = 27 (14 from tumor patients and 13 from epilepsy patients, see Materials and Methods; young mouse n = 35; adult mouse n = 11). (B) Inactivation time constant. (mean ± SEM) 2.2±0.3 ms human, 1.9±0.1 ms for young mouse synapses and 2.1±0.3 ms for adult mouse synapses, p>0.05; human n = 27 (14 from tumor patients and 13 from epilepsy patients, see Materials and Methods; young mouse n = 35; adult mouse n = 11). (C) Membrane time constant measured from membrane potential deflection upon short current pulse injection (mean ± SEM) 19.5±1.0 ms human, 25.1±0.4 ms for young mouse synapses and 24.6±2.0 ms for adult mouse synapses, adult and young mice have significantly higher membrane time constants than humans (p<0.05); human n = 27; young mouse n = 27; adult mouse n = 15. (D) Membrane time constant, as in a leaky integrate-and-fire neuron model, (mean ± SEM) 28±3 ms human, 22±1 ms for young mouse synapses and 42±4 ms for adult mouse synapses, adult mice have significantly higher membrane time constants than humans and young mice (p<0.05 and p<0.001, respectively); human n = 27 (14 from tumor patients and 13 from epilepsy patients, see Materials and Methods; young mouse n = 35; adult mouse n = 11). (E) More examples of the time course of synaptic depression on a 30 Hz train of EPSPs. 8 pulses+1 recovery pulse 500 ms after the 8th pulse for mouse connections and 300 ms after the 8th pulse for a human connection (of the n = 27 pairs measured in human slices, six were probed with 500 ms and 21 with 300 ms between the 8th and 9th pulse). (TIF) [file pbio.1002007.s001.tif]

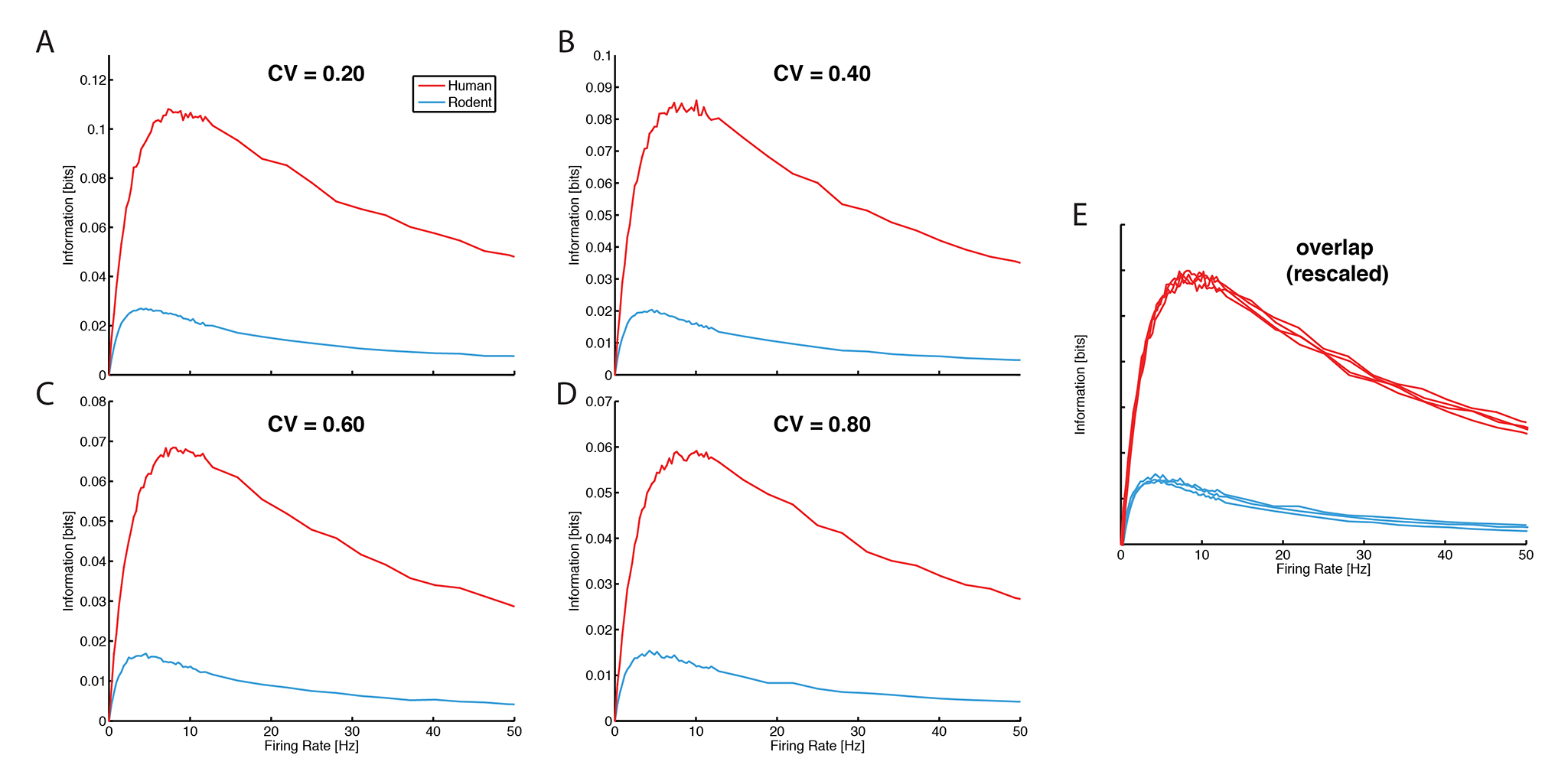

Supplement: Figure S2 — Different values of CV of the simulated quantal content (i.e., 0.2, 0.4, 0.6, 0.8) only scales the mutual information. From (E), we can conclude that in the model the CV parameter has no qualitative effect. Changing its value results in a scaling effect only. The figure was obtained with a smaller number of simulated Poisson spikes, it therefore appears noisier than Figure 3G and 3H. (TIF) [file pbio.1002007.s002.tif]

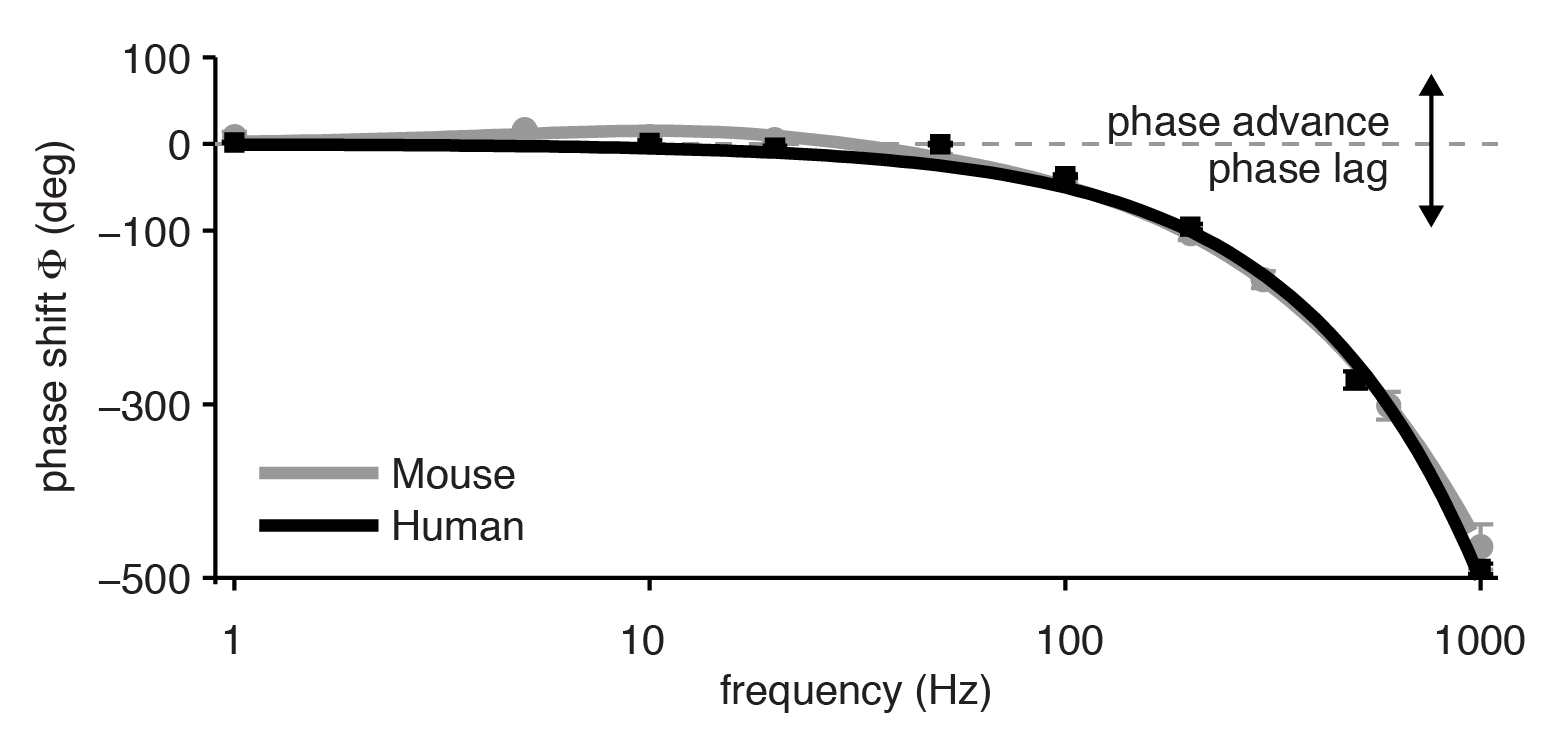

Supplement: Figure S3 — When the dynamical response properties of human (black) and rodent (gray) pyramidal neurons are analyzed in terms of phase Φ( f ), instead of response magnitude ( Figure 4 ), very similar profiles in human and mouse neurons are observed across the Fourier frequencies. Mouse neurons revealed a more prominent low Fourier frequency phase advance than human neurons, as a direct consequence of more prominent spike-frequency adaptation. (TIF) [file pbio.1002007.s003.tif]

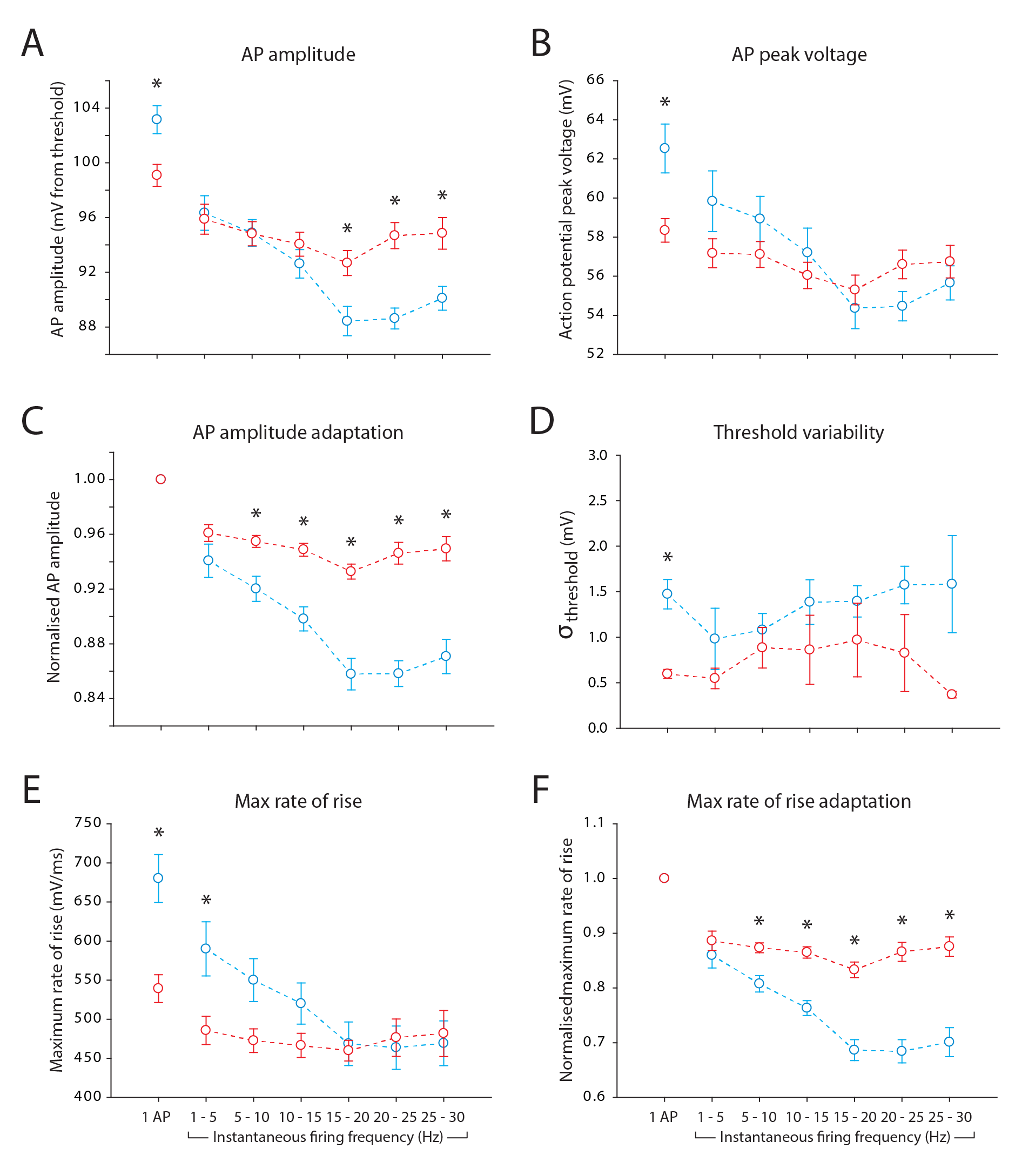

Supplement: Figure S4 — Action potential parameter comparisons for mouse and human neurons. (A–F) Quantification of various AP features of human (red) and mouse (blue) neurons, displayed versus firing frequency. Data are presented as means ± SEM. for single APs, and APs fired in trains binned in 5 Hz bins according to instantaneous firing frequency. Asterisks indicate p<0.005. (A) AP amplitude, in mV from threshold. (B) AP peak voltage. (C) AP amplitude adaptation. (D) Threshold variability, presented as the standard deviation of AP thresholds. (E) Maximum rate of rise. (F) Maximum rate of rise adaptation. For more details on how AP features were calculated, see Methods. (TIF) [file pbio.1002007.s004.tif]

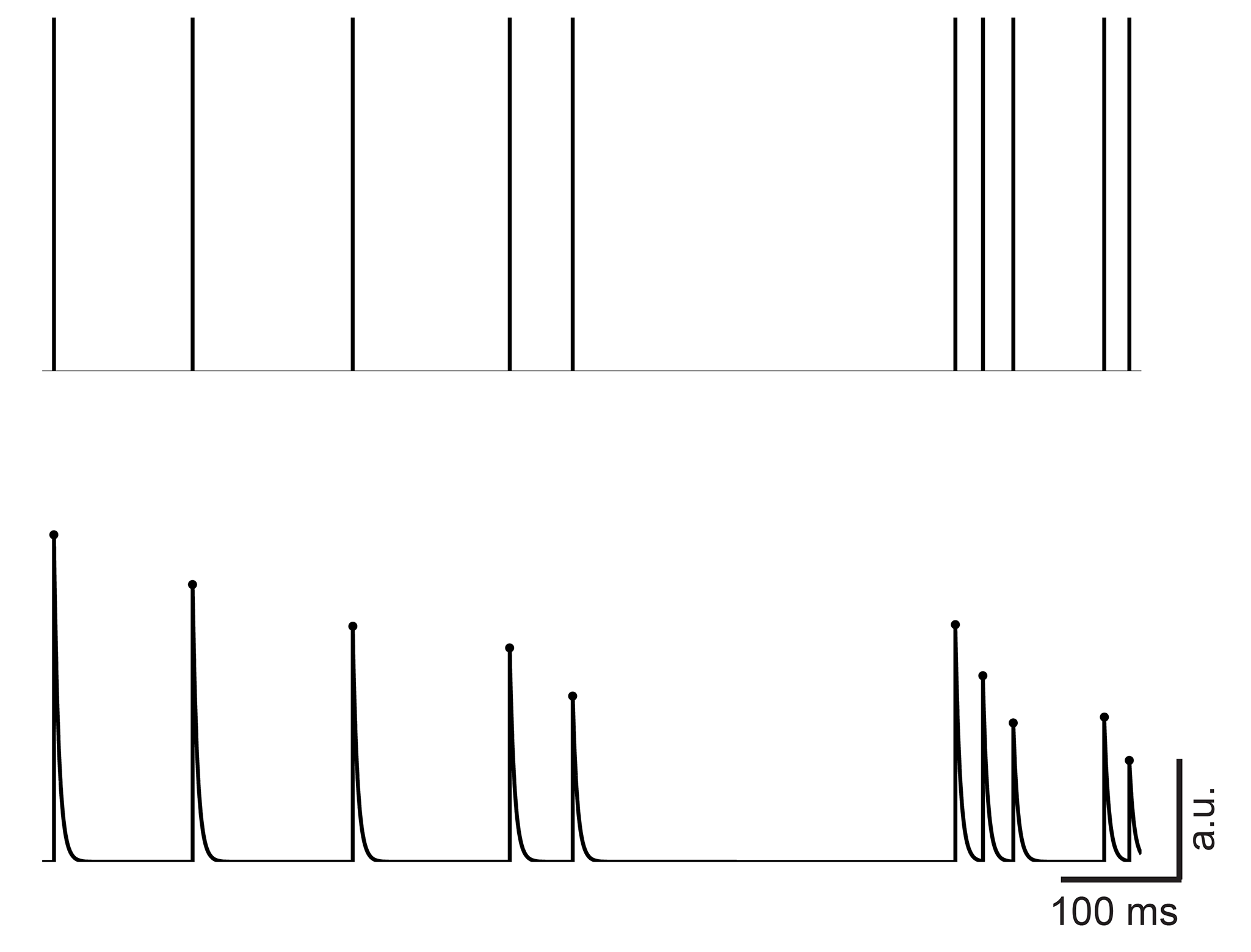

Supplement: Figure S5 — Average 10 Hz 10-spike long Poisson train is depicted, together with resulting synapse raw responses, for the deterministic Tsodyks-Markram model. (TIF) [file pbio.1002007.s005.tif]

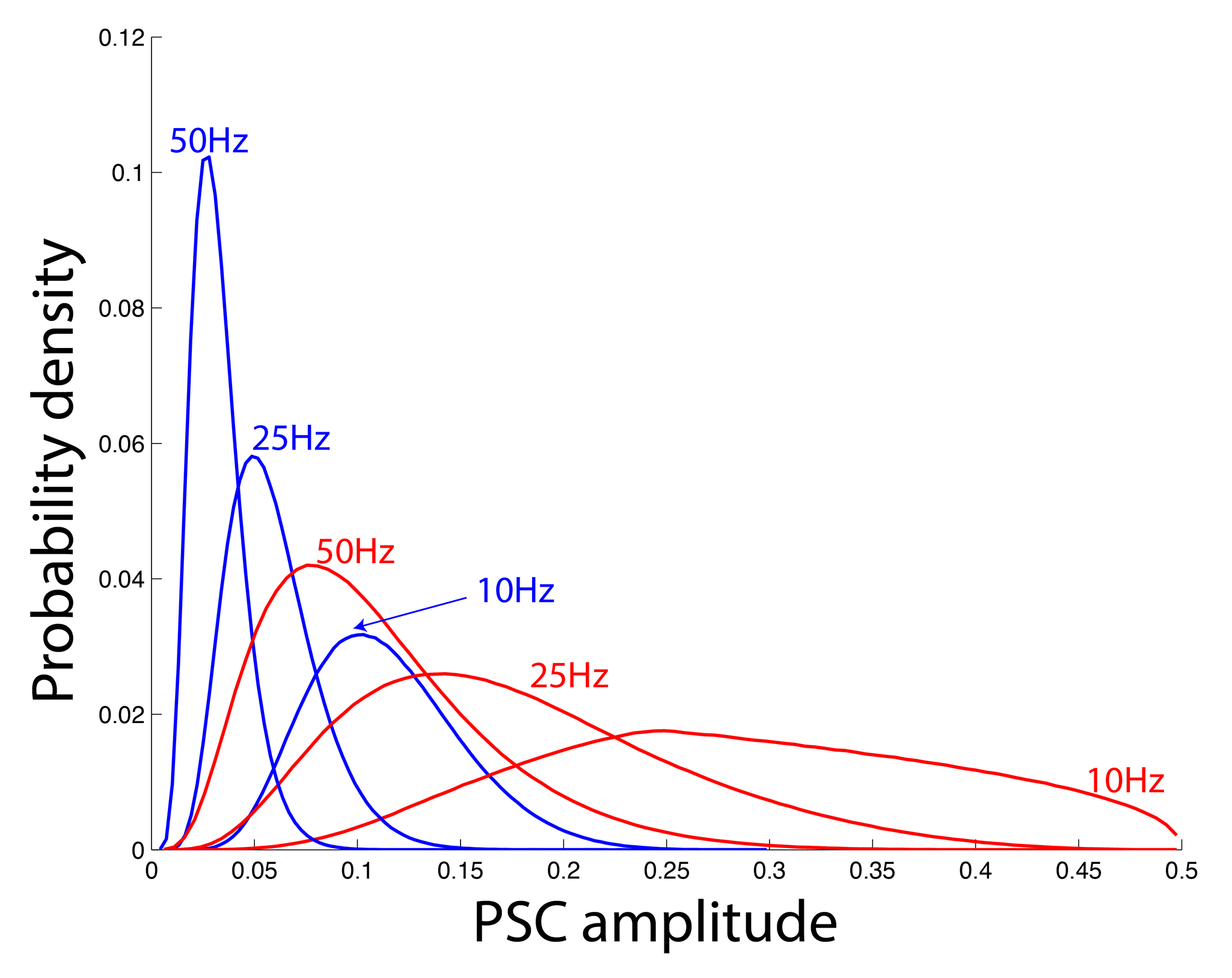

Supplement: Figure S6 — Increasing (average) presynaptic spiking rate, the postsynaptic current (PSC) histogram shifts to weaker peak values (i.e., short-term depression). (TIF) [file pbio.1002007.s006.tif]
