# Supplementary material for: High Bandwidth Synaptic Communication and Frequency Tracking in Human Neocortex
Source: PLoS Biol. 2014 Nov 25;12(11):e1002007. doi: 10.1371/journal.pbio.1002007 (PMC4244038; doi:10.1371/journal.pbio.1002007)
Supplement: Table S1 — Parameters derived by the Tsodyks-Markram model for dynamically depressing synapses. (DOCX) [file pbio.1002007.s007.docx]

**Supplementary Table 1. Tsodyks- Markram model for dynamically depressing synapses**

|  | Human (n=27) | Young mouse (n=35) | Adult mouse (n=11) | p _(Human vs Young mouse)_ | p _(Human vs Adult mouse)_ | p _(Young vs Adult mouse)_ |
| --- | --- | --- | --- | --- | --- | --- |
| A (mV) | 3,7$\pm$  0,5 | 5,6$\pm$  1,1 | 1,6$\pm$  0,4 | n.s. | n.s. | n.s. |
| U | 0,45$\pm$  0,03 | 0,25$\pm$  0,02 | 0,29$\pm$  0,03 | p<0.001 | p<0.05 | n.s. |
| Tau rec (ms) | 144$\pm$  13 | 536$\pm$  40 | 483$\pm$  91 | p<0.001 | p<0.001 | n.s. |
| Tau mem (ms) | 28$\pm$  3 | 22$\pm$  1 | 42$\pm$  4 | n.s. | p<0.05 | p<0.001 |
| Tau Inac (ms) | 2,2$\pm$  0,3 | 1,9$\pm$  0,1 | 2,1$\pm$  0,3 | n.s. | n.s. | n.s. |
