# Supplementary material for: High Bandwidth Synaptic Communication and Frequency Tracking in Human Neocortex
Source: PLoS Biol. 2014 Nov 25;12(11):e1002007. doi: 10.1371/journal.pbio.1002007 (PMC4244038; doi:10.1371/journal.pbio.1002007)
Supplement: Table S2 — Kinetic parameters from EPSPs. (DOCX) [file pbio.1002007.s008.docx]

**Supplementary Table 2.EPSP kinetics**

|  | Human (n=27) | Young mouse (n=35) | Adult mouse (n=11) | p _(Human vs Young mouse)_ | p _(Human vs Adult mouse)_ | p _(Young vs Adult mouse)_ |
| --- | --- | --- | --- | --- | --- | --- |
| 9^th^ / 1^st^ EPSP | 0,94$\pm$  0,03 | 0,67$\pm$  0,03 | 0,72$\pm$  0,06 | p<0.001 | p<0.01 | n.s. |
| 8^th^ / 1^st^ EPSP | 0,38$\pm$  0,03 | 0,44$\pm$  0,05 | 0,30$\pm$  0,04 | n.s. | n.s. | n.s. |
| Max Amplitude (mV) | 1,3$\pm$  0,2 | 1,5$\pm$  0,2 | 1,9$\pm$  0,4 | n.s. | n.s. | n.s. |
| Rise time (ms) | 1,7$\pm$  0,3 | 1,4$\pm$  0,1 | 1,9$\pm$  0,1 | n.s. | n.s. | n.s. |
| Decay time (ms) | 32$\pm$2 | 34$\pm$2 | 59$\pm$7 | n.s. | p<0.001 | p<0.001 |
| Decay time const. (ms) | 41$\pm$7 | 90$\pm$22 | 62$\pm$16 | n.s. | n.s. | n.s. |
