# Supplementary material for: High Bandwidth Synaptic Communication and Frequency Tracking in Human Neocortex
Source: PLoS Biol. 2014 Nov 25;12(11):e1002007. doi: 10.1371/journal.pbio.1002007 (PMC4244038; doi:10.1371/journal.pbio.1002007)
Supplement: Table S3 — Intrinsic cell properties, calculated from resting membrane potential and steady-state response to hyperpolarizing current injection pulse. (DOCX) [file pbio.1002007.s009.docx]

**Supplementary Table 3. Intrinsic cell properties**

|  | Human (n=27) | Young mouse (n=45) | Adult mouse (n=26) | p _(Human vs Young mouse)_ | p _(Human vs Adult mouse)_ | p _(Young vs Adult mouse)_ |
| --- | --- | --- | --- | --- | --- | --- |
| Input resistance (mΩ) | 70$\pm$6 | 84$\pm$3 | 102$\pm$7 | n.s. | p<0.001 | n.s. |
| Rest Vm | -73.2$\pm$  0.8 | -69.6$\pm$  0.7 | -74.5$\pm$  1.1 | p<0.05 | n.s. | p<0.001 |
