# Supplementary material for: High Bandwidth Synaptic Communication and Frequency Tracking in Human Neocortex
Source: PLoS Biol. 2014 Nov 25;12(11):e1002007. doi: 10.1371/journal.pbio.1002007 (PMC4244038; doi:10.1371/journal.pbio.1002007)
Supplement: Methods S1 — Extension of the information theory mathematical framework to dynamic synapses. (DOCX) [file pbio.1002007.s014.docx]

**Supplementary methods:**

We followed the standard theoretical framework, first proposed by Fuhrmann, Segev, Markram, and Tsodyks in 2002, for quantifying the efficiency of temporal coding by a short-term depressing (model) synapse.

Specifically, we studied the **mutual information** **I(PSC, ISI)** between a train of presynaptic spikes (i.e., a series of inter-spike intervals, **ISIs**) and the amplitude of the postsynaptic response (i.e., the **PSC** peak amplitude). **We then ask how much the knowledge/observation of PSC reduces the uncertainty on the ISIs preceding it:**

I(PSC, ISI) = H(PSC) - H(PSC / ISI)

As in the case of characterizing a communication channel, information theory makes explicit neither a particular information code, nor a decoding strategy. Instead, a “generator” (i.e., a transmitter), with large uncertainty (i.e., randomness), is chosen as the source of the ISIs. For this generator, we employed the exponential distribution, with mean 1/λ (i.e., known to be the maximum entropy distribution). As cortical spikes trains in vivo are also very frequently assimilated to Poisson processes (i.e., their ISI is exponentially distributed), our choice is also by physiology (Figure S5).

In the plot included above, a (average 10Hz) 10-spikes long Poisson train is depicted, together with resulting synapse raw responses, for the deterministic TM model.

For this model, the conditional entropy H(PSP / ISI) is zero, because whenever a sequence of ISIs is given, there is no uncertainty (i.e., determinism) in predicting the value of a PSP. Then the mutual information is mathematically estimated by the entropy H(PSC) of the PSC, which requires computing the histogram of the PSCs (i.e., an estimate of the probability density function) over a very large number of realizations.

As shown in the next plot, below, it is observed that for increasing (average) presynaptic spiking rate, the PSC histogram shifts to weaker peak values (i.e., short-term depression). However, due of the diversity of the best-fit model parameters, the distributions have considerably different variances, with the ones corresponding to human cortical synapses (red) remaining anyway wider than those corresponding to rodent synapses (blue). Wider variance implies higher uncertainty and thus higher entropy H(PSC) and ultimately mutual information (Figure S6).

Fuhrmann, however, recommended a more realistic quantification of mutual information, consisting in excluding the synaptic failures from the analysis. Although a failure is very much informative, the postsynaptic neuron cannot know that a presynaptic spike occurred. Then, the mutual information should be evaluated not between the ISIs and the PSCs, but instead between the ISIs and the PRCs, conditional to the lack of a failure. For this, we followed closed Fuhrmann et al., (2002) and we refer to their method section: very similar consideration applies.

We would like to finally note that such a particular definition of information transfer is meaningful only for short-term plastic synapses and, consequently, for **history-dependent PSCs**. An ideal synapse that transmits spikes always by identical PSC amplitude, would clearly not allow an observer to reconstruct the temporal structure of the presynaptic spike-train, by the sole observation of the PSCs (i.e., the mutual information would be zero in our framework).

In our work instead, we aimed at (information) theoretically exploring the performance of any presynaptic spike times inference, from the observations of the amplitudes of each PSC (e.g., intuitively, very small PSC suggests a very short preceding ISI, due to short-term depression).

**Relevant observations on Figure 5 and Figure S4:**

Traces with resting membrane potential >-60 mV were excluded from analysis. APs where linear fit to obtain onset rapidity had R^2 value <0.95 were excluded from analysis. Onset rapidity was defined as the slope of a linear fit to the AP phase plot (dV/dt vs V, with unit 1/ms) at dV/dt = 30 mV/ms (fits with R^2 values <.95 were excluded from analysis).  (Rationale: since onset rapidity is measured in linear phase of phase plot, lower Rsq values are usually an indication of noise and indeed gave either extremely low or off-the-chart high onset rapidity values).
